# Frequency of Follow-Up Assessment for Post-Intensive Care Syndrome Among Alert and Non-Delirious Critically Ill Patients

**DOI:** 10.7759/cureus.32027

**Published:** 2022-11-29

**Authors:** Rachel A Hadler, Franklin Dexter, Brian Gu

**Affiliations:** 1 Anesthesia, University of Iowa, Iowa City, USA

**Keywords:** chart review, observational cohort study, psychological distress in caregiving, icu delirium, intensive care unit stay, post-intensive care syndrome

## Abstract

Introduction: Many patients surviving critical illness develop post-intensive care syndrome, a constellation of psychological, physical, and cognitive symptoms which can have long-term consequences. Physicians and nurses at our large rural teaching hospital treat many of the critically ill patients in the state. Our focus has been the subset of these critically ill patients who were alert and not delirious for multiple consecutive days. The goal of our retrospective cohort study was to estimate the percentage of the patients with multiple intensive care unit days alert and not delirious who had follow-up assessments for post-intensive care syndrome within 15 months.

Methods: The inclusion criteria for the case series of randomly selected patients were: adults defined as patients aged >17 years on the date of hospital admission between October 2014 and December 2020, present in a critical care unit at noon one day and continually so for another 48 hours, and for that interval, ≥≥48 hours had every Riker sedation-agitation scale “4, calm and cooperative,” as well as either all Confusion Assessment Method for the Intensive Care Unit scores negative (i.e., no delirium) or Delirium Observation Screening Scale <3 (i.e., no delirium). Each patient was then categorized as having a full one-year follow-up if there was an encounter at our hospital between 12 and 15 months after the last date meeting study inclusion criteria. All follow-up appointments completed within 15 months of the index intensive care unit stay were screened for systematic assessment for psychological and cognitive sequelae of critical illness.

Results: From a manual chart review of 366 records, 73 patients were found with follow-up ≥≥12 months. There were 21% (15/73) of the patients assessed for post-intensive care syndrome sequelae (99% confidence interval 10%-35%).

Conclusions: The fact that far fewer than half the patients had documented assessments suggests that retrospective studies should not be used to judge the incidence of post-intensive care syndrome at our hospital. Prospective observational studies would be needed to judge outcomes among critically ill patients with multiple consecutive days of alert and without delirium.

## Introduction

Many patients surviving critical illness develop post-intensive care syndrome, a constellation of psychological, physical, and cognitive symptoms which can have long-term consequences [1,2]. Some hospitals have developed post-intensive care clinics as a strategy for following and assessing high-risk patients, analogous to the follow-up by neonatologists of infants discharged from neonatal intensive care units. To our knowledge, no organized options for systematic post-intensive care follow-up are available in our state. As such, survivors of critical illness are dependent upon other clinicians, less familiar with critical illness screening, to recognize post-intensive care syndrome. There is no International Classification of Disease diagnosis code for post-intensive care unit syndrome [3].

Physicians and nurses at our large rural teaching hospital treat many of the critically ill patients in the state. Our research focus has been the study of the subset of these critically ill patients who were alert and not delirious (i.e., calm, cooperative, and conversant) for multiple consecutive days [4]. The goal of the current retrospective cohort study was to estimate the percentage of the patients with multiple intensive care unit days alert and not delirious, who after being discharged alive, had follow-up assessment for post-intensive care syndrome within 15 months.

## Materials and methods

Our retrospective cohort study was approved by the University of Iowa Institutional Review Board with a waiver of written informed consent.

Adult patients were selected at random from an existing database of intensive care unit stays with the patient continually alert and not delirious for several days in the surgical or cardiovascular intensive care unit between October 1, 2014 (when electronic health record data was complete) and December 31, 2020. The uniformly distributed random numbers used for the rank order selection of patients were generated using the Mersenne Twister algorithm in Excel (Microsoft 365, Redmond, USA). The study end date was the latest end of a quarter (i.e., December 31) that was at least 15 months before the start of the current study (i.e., April 1, 2022) to ensure that all follow-up encounters within one year of the intensive care unit admission were captured. The inclusion criteria for the study patients were: adults defined as patients aged >17 years on the date of hospital admission, present in a critical care unit at noon one day and continually so for another 48 hours, and for that interval, ≥≥48 hours had every Riker sedation-agitation scale “4, calm and cooperative,” as well as either all Confusion Assessment Method for the Intensive Care Unit scores negative (i.e., no delirium) or Delirium Observation Screening Scale <3 (i.e., no delirium). Thus, throughout the paper, when we refer to the patients as being “alert and not delirious,” we mean that whenever the patient was instructed to answer questions, they did so without suggestion of delirium. Although there were no data on how often patients were sleeping and woken to answer questions or assessment was deferred, alertness was assessed several times daily, and delirium assessments were made at least daily. If the Riker scale were missing, or if both the Confusion Assessment Method and Delirium Observation Screening Scale were missing, then the criteria were considered not satisfied. Importantly, our criteria included only the first 48-hour period meeting criteria for each patient. Intubation was not considered to be an exclusion criterion if the documentation, as above, was consistent with the patient’s ability to take part fully in the delirium assessment, and the patient was determined not to be delirious.

Manual chart review of the selected records was performed using the Epic electronic health record. Each patient was categorized as having full, one-year follow-up if there was an encounter at our hospital between 12 and 15 months after the last date meeting study inclusion criteria. All appointments completed within 15 months of the index intensive care unit stay were screened for systematic assessment of psychological or cognitive sequelae of critical illness using a validated instrument (e.g., Patient Health Questionnaire-9 or General Anxiety Disorder-7). Demographic variables were obtained from the electronic health record. Major comorbidities were captured from the discharge summary and recorded using Centers for Medicare and Medicaid Services codes. The district of the patient’s home residence was recorded as defined by the Iowa Hospital Association.

To facilitate power calculations, 53 patient records were initially selected at random, with 5.66% (3/53) having had an assessment within a 15-month follow-up. A new set of patient records meeting inclusion criteria were then selected at random, without duplication, until there were 73 patients identified with complete follow-up. The sample size of 73 was chosen to achieve 90% statistical power to detect the two-sided difference with a Type I error rate of 0.01 between 20% and the estimate of 5.66% (StatXact-12, Cytel, Inc., Cambridge, MA).

## Results

Of 366 patient records screened to obtain the studied 73 patients, 247 did not have a full follow-up, and 46 of the 366 were excluded based on charting discrepancies. Examples of these discrepancies included the Confusion Assessment Method for the Intensive Care Unit documentation that was discordant with Delirium Observation Screening Scale documentation (e.g., CAM-ICU negative, DOSS positive) and repeat hospitalizations for the same patient. Characteristics of the 73 unique patients with full follow-up and the 247 unique patients (366-73-46) lacking full follow-up are given in Table 1. None (0%) of the 366 patients was assessed for post-traumatic stress disorder.

**Table 1:** Characteristics of the adult patients with at least two days alert and not delirious in the intensive care unit a As explained in the last paragraph of the Methods and the first paragraph of the Results, there were 366 patient records screened to obtain the studied 73 patients. There were no (0%) assessments for post-traumatic stress disorder among any of the 366 patients. b  Patients with assessment for post-intensive care syndrome were 21% among patients with complete follow-up versus 12% without full follow-up. The smaller observed percentage shows the validity of our primary endpoint. c  A larger percentage of patients without follow-up developed delirium after being alert and not delirious for at least two days in the intensive care unit. We doubt that this was meaningful because more patients also died. Among the patients without complete follow-up, there were N=66 developing delirium and N=66 who died. The same number was not a typographical error; those were the counts. d There was reliably no difference in results for the subgroup of patients residing in the region closest to the hospital, Fisher's exact test two-sided P = 0.50. e  Comparing groups based on race listed as white, Fisher’s exact test two-sided P = 0.0094. Comparing groups based on age $\geq$80 years, Fisher’s exact test P = 0.0095. There were 33/34 of the patients age $\geq$80 years who were white (i.e., the groups may have differed based on both demographic characteristics).

Characteristic	Number (%) of the 73 patients with follow-up for at least 12-months	Number (%) of the 247 patients lacking follow-up for at least 12-months
Assessment for post-traumatic stress disorder after hospital discharge	0 (0%)^a^	0 (0%)^a^
Assessed for mood disorders or new cognitive dysfunction	15 (21%)^b^	30 (12%)^b^
Assessments of mood, with clinic types in next five rows	10	16
Assessment of mood at cardiac rehabilitation	4	3
Assessment of mood at behavioral health visit	5	2
Assessment of mood at family medicine visit	1	2
Assessment of mood at neurology, neurosurgery, or neuropsychology	0	7
Assessment of mood at palliative care	0	2
Assessments of cognitive function, with clinic types in next two rows	9	16
Assessment of cognitive function at behavioral health visit	4	0
Assessment of cognitive function at neurology, neurosurgery, or neuropsychology	5	16
Outcome of patient, given in the next two rows		
Developed delirium during the rest of their hospitalization	9 (12%)^c^	66 (27%)^c^
Died during hospitalization or within 12 months post-discharge	0 (0%)^c^	66 (27%)^c^
Demographics as listed in medical record, given in the next seven rows		
Female sex	32 (44%)	104 (42%)
Residence in hospital’s health district	34 (47%)^d^	103 (42%)^d^
Major comorbidities	38 (52%)	116 (47%)
Medicare or Medicaid insurance	61 (84%)	191 (77%)
White race	71 (97%)^e^	215 (87%)^e^
Age $\geq$ 65 years	29 (40%)	130 (53%)
Age $\geq$ 80 years	2 (3%)^e^	32 (13%)^e^

There were 21% (15/73) patients assessed for components of post-intensive care syndrome during follow-up (99% Clopper-Pearson exact confidence interval 10% to 35%) (Table 1). Among the 15 patients, there were 10 assessments of mood and nine assessments of cognitive function. The results suggested elements of post-intensive care syndrome in 40% of assessed patients (6/15). After being alert and not delirious in the intensive care unit, 12% of patients (9/73) developed delirium during the rest of their hospitalization.

The pilot tranche of patient records had 5.66% (3/53) assessment for post-intensive care syndrome. The comparator would be all patients in the study population, 12.3% (45/366) with assessment. The pilot and study proportions did not differ significantly Fisher’s exact test P = 0.24.

## Discussion

At our large rural teaching hospital, approximately 20% of intensive care unit survivors received follow-up for up to 15 months following admission. Of this population, far fewer than half of these patients had documented assessments for the psychological and cognitive symptoms of post-intensive care. Although the specific risks of post-traumatic stress disorder, anxiety, depression, and cognitive changes in non-delirious, lucid patients with prolonged (>48 hours) length of stay are unknown, these patients certainly retain some likelihood of developing symptoms related to their intensive care unit stay, particularly psychological symptoms [5]. Although these findings represent practice patterns at a single hospital, they suggest that even if there were an International Classification of Diseases code for post-intensive care unit syndrome [3]; the use of administrative data to estimate incidence among these patients alert and not delirious would be unreliable. Nevertheless, there currently is no such code(s) [3]. Therefore, retrospective cohort studies reliably cannot be used to judge the incidence of post-intensive care syndrome among this unique group of patients, at least at our hospital. Instead, the prospective observational study would be needed to estimate the incidence of post-intensive care syndrome among the patients alert and without delirium for several days while in an intensive care unit.

Lack of reliable assessment of patient emotional and cognitive function acts as a barrier to testing interventions for reducing the prevalence of post-intensive care syndrome include. These assessments require direct, sensitive communication with patients as well as their completion of valid and reliable instruments. When evaluated, many of these patients are suffering from severe psychological distress [4]. Studies evaluating post-intensive care syndrome have been largely retrospective [2,6-7], reliant upon highly variable recall of events in the intensive care unit [8,9]. Our observation that approximately 12% of the patients developed delirium suggests that intensive care unit patients alert and not delirious probably can be screened and treated for distress with acceptable recall and low dropout rates. Future prospective work is needed to assess how distress evolves over time and whether intensive care unit distress is predictive of the psychological and/or cognitive sequelae of critical illness.

Data shown in the last several rows of Table 1 suggest that patients’ age and race may have been associated with follow-up for symptoms of post-intensive care syndrome. We do not know if these are reliable differences (e.g., if older patients had more follow-up). However, if so, the observations suggest further that the 15 patients with complete follow-up and assessment for post-intensive care syndrome should not be considered representative of the other 351 patients’ risk for the syndrome. (The 351 = 366 total minus the 15 patients.)

Study limitations include that the present study is a single-center retrospective cohort investigation. We only counted assessments of mood and cognitive function that were documented in the electronic health record. The incidence of assessment might be greater (e.g., some patients may have had formal psychological assessment at other healthcare facilities). However, we deliberately did not rely on the Epic Care Everywhere Network as the information contained is not always complete or representative of all the care a patient is receiving. Based on our power calculations, increasing the sample size of patients with follow-up for 15 months was unnecessary. Our goal was to have complete medical records, including all patient assessments with validated psychological instruments. Because our study only examined the follow-up of patients from one hospital, limited inferences can be made about other institutions’ practices and follow-up patterns. However, other data suggest too that without an intensive care unit follow-up program, fewer than half the patients may have assessment for post-intensive care syndrome. In a New York City hospital with critical care recovery clinic, the percentage of patients meeting referral criteria who then completed evaluation in 2020 was 37% (45/121) [10]. In The Netherlands, with general practitioner follow-up, national survey showed that only 14% (38/266) of the physicians were aware of intensive care unit follow-up programs [11]. In Australia, national survey of intensive care units found that only 5% (5/107) had follow-up clinics or routine telephone follow-up [12].

## Conclusions

Rates of assessment for cognitive and psychiatric sequelae of critical illness at our large teaching hospital in a rural state were low, with far fewer than half of the patients. Therefore, prospective observational studies would be needed to judge incidences of outcomes among critically ill patients with multiple consecutive days of alert and without delirium.
